# Long Noncoding RNA Expression during Human B-Cell Development

**DOI:** 10.1371/journal.pone.0138236

**Published:** 2015-09-22

**Authors:** Andreas Petri, Karen Dybkær, Martin Bøgsted, Charlotte Albæk Thrue, Peter H. Hagedorn, Alexander Schmitz, Julie Støve Bødker, Hans Erik Johnsen, Sakari Kauppinen

**Affiliations:** 1 Center for RNA Medicine, Department of Clinical Medicine, Aalborg University, Copenhagen, Denmark; 2 Department of Haematology, Aalborg University Hospital, Aalborg, Denmark; 3 Department of Clinical Medicine, Aalborg University, Aalborg, Denmark; 4 Clinical Cancer Research Center, Aalborg University Hospital, Aalborg, Denmark; Sun Yat-sen University, CHINA

## Abstract

Long noncoding RNAs (lncRNAs) have emerged as important regulators of diverse cellular processes, but their roles in the developing immune system are poorly understood. In this study, we analysed lncRNA expression during human B-cell development by array-based expression profiling of eleven distinct flow-sorted B-cell subsets, comprising pre-B1, pre-B2, immature, naive, memory, and plasma cells from bone marrow biopsies (n = 7), and naive, centroblast, centrocyte, memory, and plasmablast cells from tonsil tissue samples (n = 6), respectively. A remapping strategy was used to assign the array probes to 37630 gene-level probe sets, reflecting recent updates in genomic and transcriptomic databases, which enabled expression profiling of 19579 long noncoding RNAs, comprising 3947 antisense RNAs, 5277 lincRNAs, 7625 pseudogenes, and 2730 additional lncRNAs. As a first step towards inferring the functions of the identified lncRNAs in developing B-cells, we analysed their co-expression with well-characterized protein-coding genes, a method known as “guilt by association”. By using weighted gene co-expression network analysis, we identified 272 lincRNAs, 471 antisense RNAs, 376 pseudogene RNAs, and 64 lncRNAs within seven sub-networks associated with distinct stages of B-cell development, such as early B-cell development, B-cell proliferation, affinity maturation of antibody, and terminal differentiation. These data provide an important resource for future studies on the functions of lncRNAs in development of the adaptive immune response, and the pathogenesis of B-cell malignancies that originate from distinct B-cell subpopulations.

## Introduction

Recent data implies that the mammalian genome is pervasively transcribed and encodes thousands of long noncoding RNAs (lncRNAs) that play distinct and specialized roles in numerous biological processes [1–6] and many diseases [7–11]. LncRNAs lack a significant open reading frame and comprise an expanding inventory of noncoding RNAs (ncRNAs) that are longer than 200 nucleotides in length, such as long intergenic ncRNAs (lincRNAs), long intronic ncRNAs, antisense RNAs, pseudogene RNAs, and transcribed ultraconserved regions [12]. Antisense transcripts are encoded on the opposite strand relative to their sense gene and they constitute a functionally diverse class of molecules that can modulate nearly all stages of gene expression (reviewed in ref [13]). The type of overlap displayed between the sense and antisense transcript can be used to further divide this sub-class into head-to-head overlapping, where the 5’ ends of the sense-antisense RNAs overlap, fully-overlapping, where the antisense transcript is fully embedded in the sense transcript, and tail-to-tail, where the 3’ ends overlap [14]. LincRNAs do not overlap with other genes and this characteristic has facilitated genetic loss-of-function studies [1], but apart from this they share many characteristics with other lncRNA classes that appear as modular scaffolds, combining distinct domains that can interact with DNA, RNA, or protein [15–17]. Although the genomic organization of antisense RNAs and lincRNAs might suggest a functional distinction into cis- and trans-acting lncRNAs, respectively, this is not always true and there are examples of trans-acting antisense RNAs [18] as well as cis-acting lincRNAs [19]. Pseudogenes constitute a class of genes that are copies of protein-coding genes, but due to accumulation of disabling mutations, the genes have lost their protein-coding potential. Thus, pseudogenes can give rise to ncRNA transcripts, whose expression have been linked to regulation of expression of their protein-coding counterpart [20].

B-cells develop from the common lymphoid progenitor cells in the bone marrow and the initial antigen-independent phase is characterized by immunoglobulin gene rearrangements through action of the RAG1 (recombination-activating gene 1)-RAG2 protein complex [21]. Once a functional B-cell receptor has been formed and B-cells have matured, the naive B-cells acquire the ability to circulate and thereby patrol the secondary lymphoid organs for cognate antigens. Upon antigen exposure within the germinal center (GC), the activated centrocyte differentiates into a rapidly proliferating centroblast that undergoes affinity maturation of the B-cell receptor (BCR) [22]. Expression of the B-cell lymphoma 6 (BCL6) gene in the centroblasts enables tolerance of DNA breaks and high proliferation rates that would otherwise induce apoptosis [23]. Further differentiation results in two long-lived B-cell populations: the memory B cells and antibody-secreting plasma cells.

While the roles of transcription factors and miRNAs in B-cell development have been extensively studied [24,25], our understanding about the functions of lncRNAs in B-cell lymphopoiesis is still limited [26–28]. Here, we describe exon array-based analysis of lncRNA expression in developing B-cell subsets isolated by flow cytometry-based sorting from human tonsils and bone marrow, respectively. The array probes were reorganized into gene-specific probe sets using updated genome information, gene models and annotation [29], and by using weighted gene co-expression network analysis [30] on the expression profiles, we identified several lncRNAs embedded in well-defined gene networks involved in specific stages of human B-cell development.

## Materials and Methods

### Collection of tonsils and bone marrow biopsies

The study was conducted in accordance with the Declaration of Helsinki, and all normal tissue samples were collected with written informed consent from each patient, in accordance with the MSCNET research protocol that was reviewed and approved by the health ethics committee for the North Denmark Region (Approval N-20080062MCH). Tonsils were collected from six patients during routine tonsillectomy as previously described [31], and bone marrow tissue was obtained by physical scraping of the medulla from seven patients undergoing cardiac surgery as described [32].

### Isolation of B-cell subsets from tonsils and bone marrow by flow cytometry

Mononuclear cells were isolated from tonsils and bone marrow and prepared for multiparametric flow cytometry using an optimized and validated protocol as previously described [32]. All cells were stained for CD10, CD20, CD27, CD38, and CD45. In addition, cells from tonsils were stained for CD3, CD44, and CXCR4, and cells from bone marrow were stained for CD19 and CD34, respectively. This allowed separation of the following distinct B-cell subsets by fluorescence-activated cell sorting (FACS): (i) naive (N(b) and N(t)) and memory (M(b) and M(t)) cells from bone marrow (b) and tonsils (t), respectively; (ii) pre-B1 (B1), pre-B2 (B2), immature (I), and end-stage antibody-producing plasma cells (PC) from bone marrow, and (iii) centrocytes (CC), centroblasts (CB), and plasmablasts (PB) from tonsils.

### Data analysis

**Fig 1:**
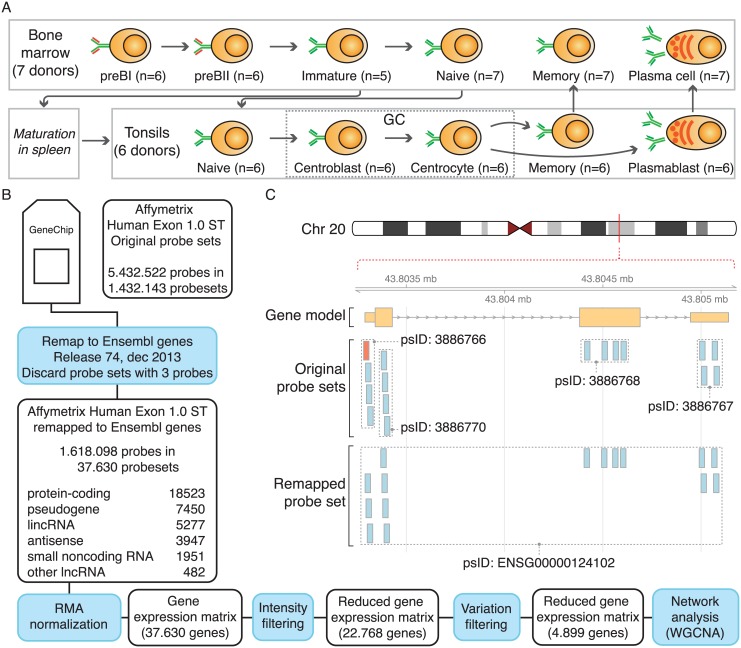
Overview of the data analysis pipeline. A) Diagram of B-cell lymphopoiesis depicting the different B-cell subpopulations isolated from bone marrow and tonsils. B) Flow diagram highlighting the different steps for processing the exon array data. C) Illustration showing the concept of working with updated annotation and remapped probe sets for gene PI3, ENSG00000124102. Four defined exon probe sets cover the three exons. The most upstream probe set contains a probe that is not fully contained in the most current gene model of PI3 (highlighted in red). The remapped probe set combines all valid PI3 probes into a single probe set.

The data acquisition and analysis are outlined in Fig 1. Data analyses and visualizations were done using R [33], BioC [34], WGCNA-package [30] and Cytoscape [35].

### Expression profiling of B-cell subsets

Expression profiling of flow-sorted B-cell subsets in human bone marrow and tonsils on Affymetrix Human Exon 1.0 ST arrays (Affymetrix, Santa Clara, CA) has been described elsewhere [32] and data have been made available at NCBI’s Gene Expression Omnibus database under accession codes GSE68878 and GSE69033. The exon array data were RMA normalized [36] using R/BioC and a custom Chip Description File (CDF), where probes were remapped into probe sets corresponding to Ensembl gene IDs (Ensembl release 74) [29,37] (as shown in Fig 1A). Probe sets containing only 3 probes were excluded from analysis.

### Assessment of coding potential

We used CPAT [38] version 1.2.1 to estimate the coding potential of transcripts encoded by genes that were detected by the remapped Affymetrix Exon array. The human prebuilt training model and hexamer frequency table distributed with the program were used. The transcript coding probabilities were summarized for each gene to give maximal coding probability, mean coding probability, and range in coding probability.

### Recursive partitioning of FACS data

From the FACS data we identified the surface markers that best separated the B-cell subsets by constructing branched binary decision trees for bone marrow and tonsil samples, respectively. In each node of a given tree, the cells were partitioned to one of two possible branches by a simple binary decision, based on the fluorescence levels of two surface markers. The trees were restricted to be maximally three nodes deep. At each node, optimal surface marker pairs and decision rules were identified as those that reduced the Gini impurity the most by an exhaustive search [39].

### Comparison of sample clustering

Hierarchical clustering was done using average linkage with Pearson’s correlation distance metric. Dendrograms resulting from sample clustering based on different gene biotypes were compared by calculating Baker's Gamma correlation coefficient as implemented in the dendextend-package [40].

### Characterization of lncRNA co-expression with their neighboring genes

Each of the 37630 genes probed on the microarray was grouped into protein-coding genes (18523), lincRNAs (5277), antisense RNAs (3947), small non-coding RNAs (1951), other lncRNAs (482), or pseudogenes (7450), based on gene biotype annotation in Ensembl [37]. For 29428 (76%) of the genes probed on the microarray, one or more neighboring genes within 1kb on the genome could be identified on either strand on the array, hereafter referred to as local pairs. For each neighbor gene pair, the genomic positions, the strand, overlapping exons or introns, and co-expression similarity were catalogued.

### Identification of gene co-expression networks

Prior to network analysis, the data were filtered to remove expression data from genes that could not be reliably detected above background and exhibited low variation across the samples. To guide the selection of intensity threshold, background probe sets were constructed that matched real probe sets in the number of probes and distribution of GC content by repeatedly sampling from the antigenomic background probes present on the exon array. The intensity threshold level was set at two standard deviations above the mean intensity of the constructed background probe sets, and genes were required to exhibit expression above this threshold for all samples in at least one B-cell subset. Furthermore, the standard deviation of gene expression across all samples was used to remove genes with low variation (standard deviation < 0.5). Weighted gene co-expression network analysis (WGCNA) [30] was used to analyze relationships between gene transcripts, essentially as described on the WGCNA website (http://www.genetics.ucla.edu/labs/horvath/CoexpressionNetwork/Rpackages/WGCNA/Tutorials/index.ht).

## Results and Discussion

### Transcriptional profiling of human B-cell lymphopoiesis

**Fig 2:**
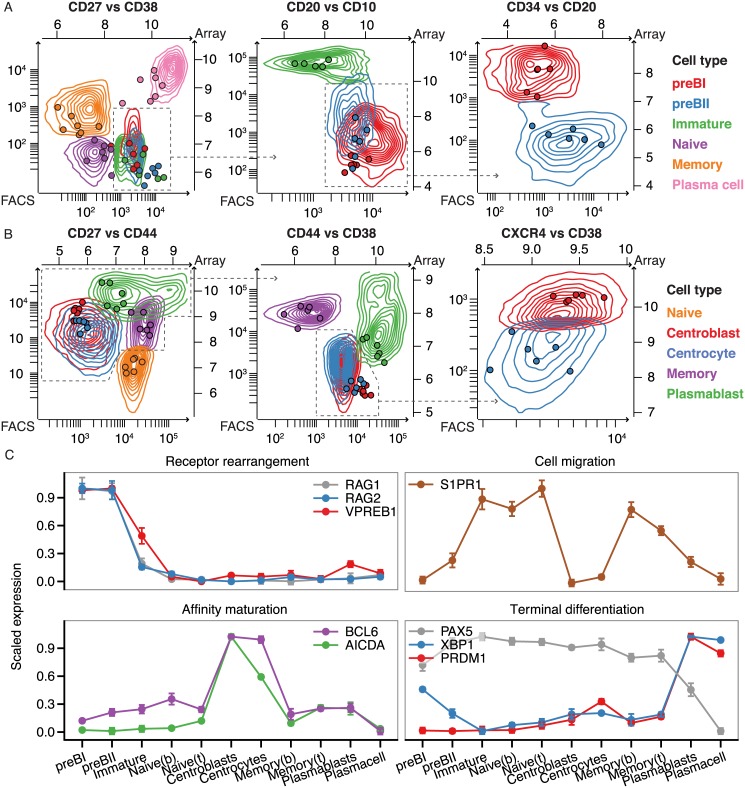
Isolation of human B-cell subsets from bone marrow and tonsils. A) Overlay of flow cytometry and array data on surface markers used for sorting of the bone marrow B-cell subsets. The contour diagrams show summary of events from the collected B-cell subsets in all samples and dots depict gene expression values (log_2_ intensities) in the individual cell samples. B) As in (A), but for tonsillar B-cell subsets and sorting markers. C) Expression profiles of selected genes, dots correspond to group means.

In this study, we isolated eleven different B-cell subsets from human sternal bone marrow and tonsil biopsies by flow cytometry [31,32], and conducted gene expression profiling using Affymetrix Human Exon 1.0 ST arrays (Fig 1A). The data were summarized using updated probe set definitions to ensure that the probe sets were consistent with recent annotations and gene models [29] (Fig 1B and 1C). This has previously been shown to improve the accuracy of gene expression profiling [41]. To validate that the flow-sorted B-cell subsets represent distinct B-cell populations, we used recursive partitioning on the multiparametric flow cytometry data to identify surface marker pairs that most effectively discriminate the sorted cell populations. The overlay of surface marker expression data on the multiparametric flow cytometry data shows that there is a high degree of concordance between marker gene expression and protein levels, and that the isolated subpopulations are well separated (Fig 2A and 2B). In addition, we find that our expression data capture several well-characterized events during B-cell development, such as expression of RAG1 and -2 along with the surrogate light chain in pre-B1 and -B2 cells and expression of S1PR1, which is necessary for immature B-cell to transfer from the bone marrow to the blood and to exit from secondary lymphoid organs [42,43]. Furthermore, we observe expression of AICDA and BCL6 in the germinal center B-cells, and reciprocal expression of transcription factor PAX5 and transcriptional repressor PRDM1 [44], as well as expression of XBP1, a key regulator of immunoglobulin secretion in terminally differentiated B-cells [45] (Fig 2C). These observations demonstrate that our expression data recapitulate key aspects of B-cell development, and can thus serve as basis for transcriptional profiling of lncRNAs in distinct B-cell subsets.

### Expression profiling of long noncoding RNAs

**Fig 3:**
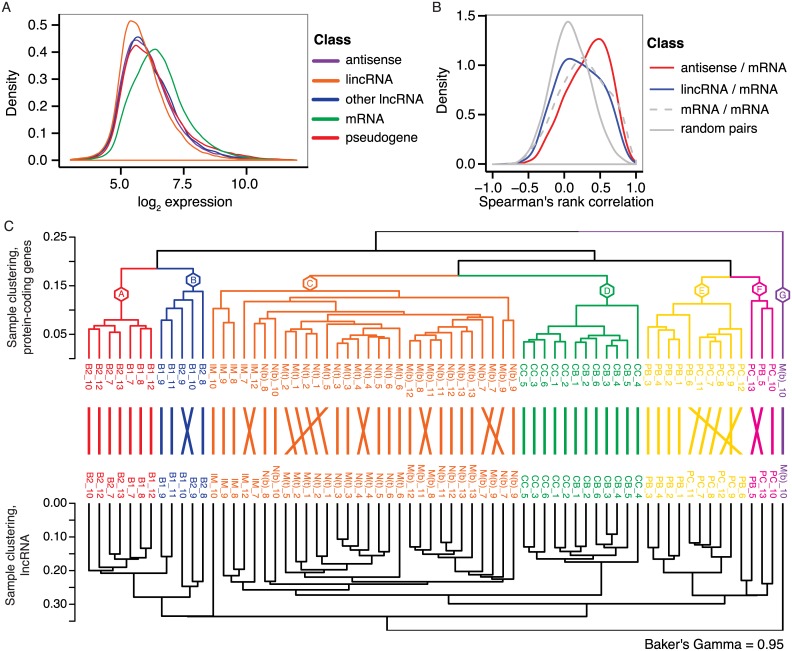
Long noncoding RNA expression in human B-cell subpopulations. A) Distribution of array-derived expression levels across all samples are shown for different gene biotype classes: Antisense, lincRNA, other lncRNA, mRNA, and pseudogene. B) Correlation of expression patterns between gene pairs located in close proximity on the genome. C) Hierarchical clustering of samples based on expression of protein-coding genes (top dendrogram) and all lncRNA classes (bottom dendrogram), respectively.

Next, we analyzed the intensity-filtered gene expression data with a focus on various classes of lncRNAs. Intensity filtering reduced the number of analyzed genes to 22768, including 2073 antisense RNAs, 1846 lincRNAs, 3475 pseudogenes, and 266 lncRNAs belonging to various classes such as 3’ overlapping ncRNAs, sense intronic and sense overlapping (collectively referred to as other lncRNAs in this manuscript). We used CPAT [38] to analyze the coding potential of transcripts derived from genes assayed by the remapped array. Transcript coding potential was summarized for each gene and used to supplement the gene biotype annotations from Ensembl. Studies employing both microarray and RNA-seq based expression profiling have reported that lncRNAs exhibit lower expression levels compared to protein-coding genes [46,47]. In accordance with these observations, we find that various classes of lncRNAs, such as lincRNAs and antisense RNAs, are expressed at lower levels compared to protein-coding mRNAs (Fig 3A). Antisense RNAs were recently shown to be important regulators of their sense partner (reviewed in ref [13]), and additionally, several lncRNAs, including lincRNAs have been shown to be involved in *cis* regulation of nearby genes [46,48,49]. Our data showed a similar trend during B-cell development (Fig 3B). Specifically, analysis of neighboring genes within 1kb showed that local antisense transcripts correlate better with corresponding sense mRNAs than local lincRNA—mRNA pairs or local mRNA—mRNA pairs. Next, we performed unsupervised hierarchical clustering of the samples based on lncRNA expression and compared to sample clustering obtained by clustering on protein-coding gene expression. We found highly similar sample grouping into distinct B-cell subsets based on expression from the two different classes (Baker’s gamma correlation of 0.95, Fig 3C), and even subdividing the lncRNAs into lincRNAs and antisense RNAs resulted in sample groupings that were very similar to protein-coding based sample clustering (Baker’s gamma correlation of 0.83 and 0.82, respectively, S1 Fig).

### Long noncoding RNA expression during human B-cell development

**Fig 4:**
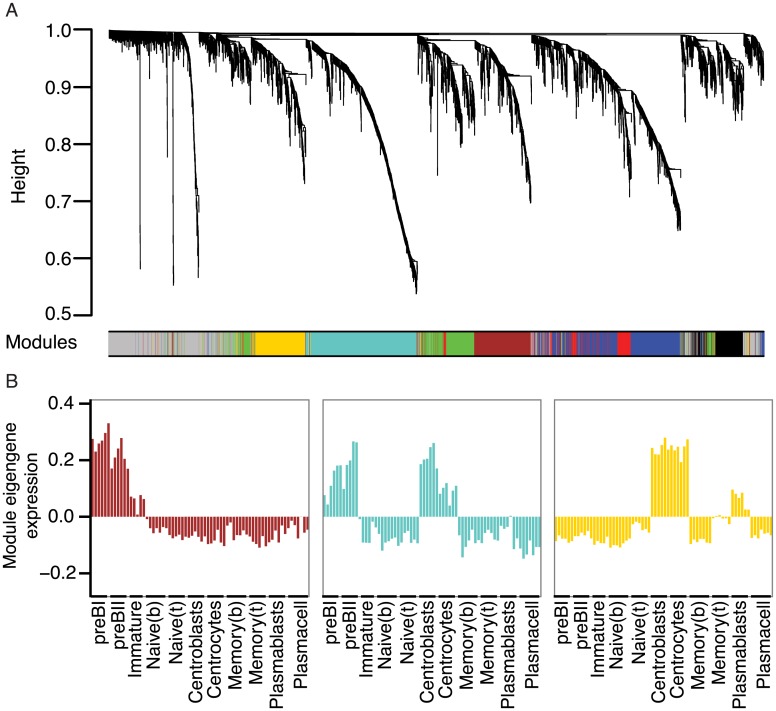
Weighted gene co-expression network analysis of human B-cell subpopulation transcriptomes. A) Cluster dendrogram showing genes grouped into distinct modules with height on the y-axis corresponding to co-expression distance between genes. B) Module expression summaries are shown with values of the components of the module eigengene (y-axis) versus microarray sample (x-axis).

**Fig 5:**
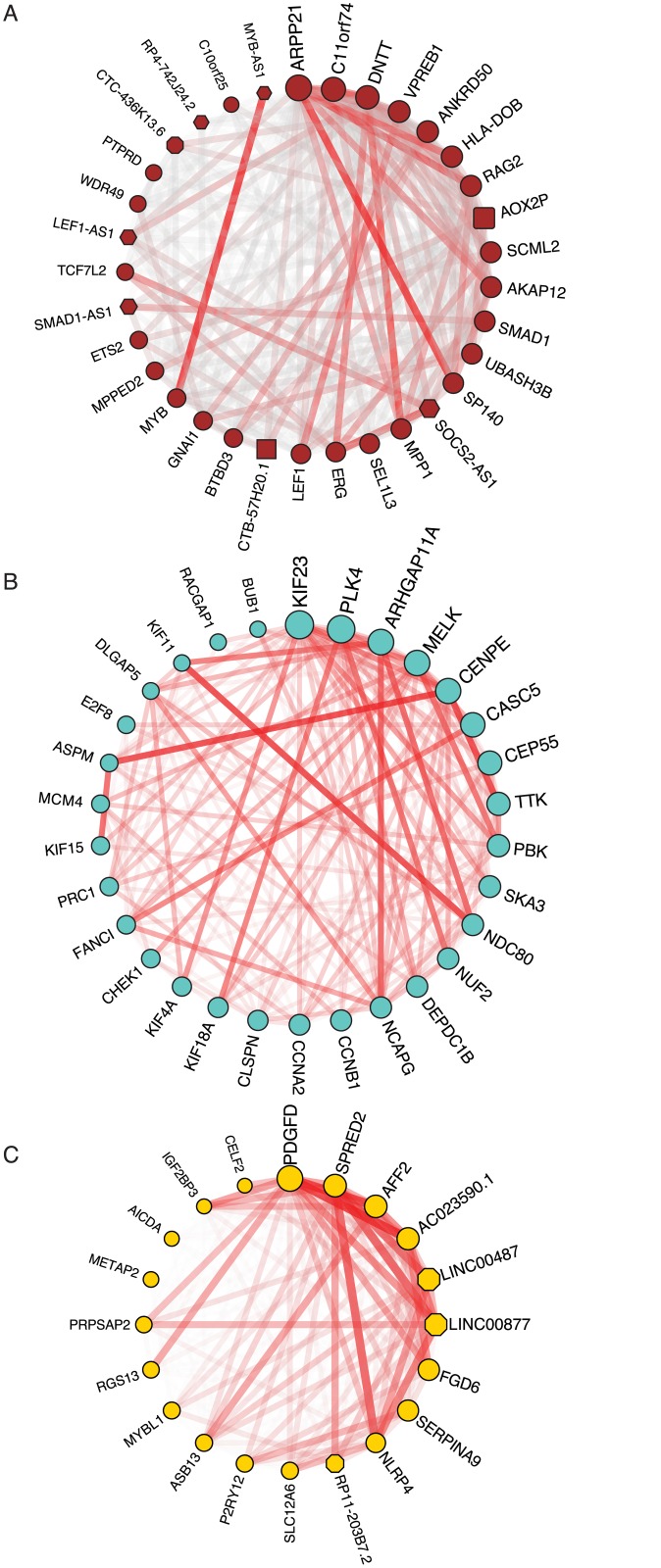
Connectivity of intramodular hub genes in three selected gene co-expression modules. A) Highly connected genes in the brown module. B) Highly connected genes in the turquoise module. C) Highly connected genes in the yellow module. The node shapes indicate gene biotype, hexagon = antisense, octagon = lincRNA, circle = protein-coding, rounded rectangle = pseudogene, and rectangle = sense overlapping. The connectivity of a gene is encoded in node size with bigger nodes meaning higher connectivity. Edge transparency and width encode gene pair adjacencies, with thicker lines and lower transparency meaning higher similarity.

The use of RNA-sequencing technologies has led to the identification of tens of thousands of lncRNAs in metazoans [47,50]. However, only a few lncRNAs have been functionally characterized. One method of predicting functions of lncRNAs from gene expression data is based on the analysis of co-expression with well-characterized protein-coding genes, a method known as guilt-by-association [15,51,52]. Co-expression alone is not sufficient to reliably assign functions to lncRNAs, but information on lncRNAs embedded in transcriptional networks associated with B-cell development provides an important starting point for functional studies. To put emphasis on genes that might partake in B-cell development, we filtered the expression data and removed genes that did not vary considerably across all samples (as described in Materials and Methods). Subsequently, we used WGCNA to describe co-expression relationships between protein-coding genes and lncRNAs and identified seven modules, which were color-coded for presentation purposes (Fig 4A and S2 Fig). The expression patterns of genes in the identified co-expression modules are summarized by the corresponding first eigengene [53] (Fig 4B and S2 Fig). Table 1 summarizes the numbers of genes annotated to different gene biotypes in each module and S1 Table lists all lncRNAs associated with the identified modules. Functional characteristics of the identified modules were analyzed by GO overrepresentation analysis and the most significantly overrepresented GO terms from each of the three ontologies (i.e. biological process, molecular function, and cellular component) are presented in Table 1. Since several studies have shown that highly connected genes (hub genes) are essential for a given gene network, we also identified hub genes in three of the identified modules (Fig 5). These modules are described in detail below.

**Table 1:** Module characteristics.

						Biological Process		Molecular Function		Cellular Component	
Module	Antisense	LincRNA	Other lncRNA	Protein coding	Pseudogene	GO term	P value	GO term	P value	GO term	P value
brown	88	65	10	281	36	regulation of immune system process	2.00E-05	signal transducer activity	5.50E-05	cytoplasmic side of membrane	0.00014
turquoise	87	35	10	672	66	mitotic cell cycle	< 1e-30	DNA binding	3.30E-11	chromosomal part	< 1e-30
yellow	50	44	6	306	53	cellular response to stimulus	0.0011	ubiquitin protein ligase binding	0.021	plasma membrane region	0.00055
blue	100	36	13	638	73	ER-nucleus signaling pathway	2.70E-21	aminoacyl-tRNA ligase activity	2.50E-05	endoplasmic reticulum	7.00E-30
red	36	30	5	205	29	organic hydroxy compound metabolic process	1.50E-05	signal transducer activity	0.0016	integral component of plasma membrane	0.0016
green	58	27	8	389	91	immune response	2.80E-06	signal transducer activity	0.0015	endosome	7.50E-07

#### Early B-cell development

Genes that are expressed during early B-cell differentiation, primarily in pre-B1 and pre-B2 cells and absent or expressed at low level during later development are shown in the brown module. Since co-expression networks are based on correlation of gene expression, the reciprocal expression profile, i.e. low or absent expression in early B-cell differentiation and high levels at later developmental stages, are also observed in the brown module (see Fig 4B and heatmaps in S2 Fig). Enrichment analysis of GO terms assigned to genes in this module show overrepresentation of the terms ‘regulation of immune system process’, ‘leukocyte activation’, ‘signal transducer activity’, and ‘nucleic acid binding transcription factor activity’ (Table 1 and S2 Table). Notably, genes assigned to ‘signal transducer activity’ include FLT3 and IL7R, both of which are growth-factor receptors required for early B-lymphopoiesis [54,55], and genes assigned to ‘nucleic acid binding transcription factor activity’ include important factors such as LEF1, MYB, and IKZF3 [56–58].

**Fig 6:**
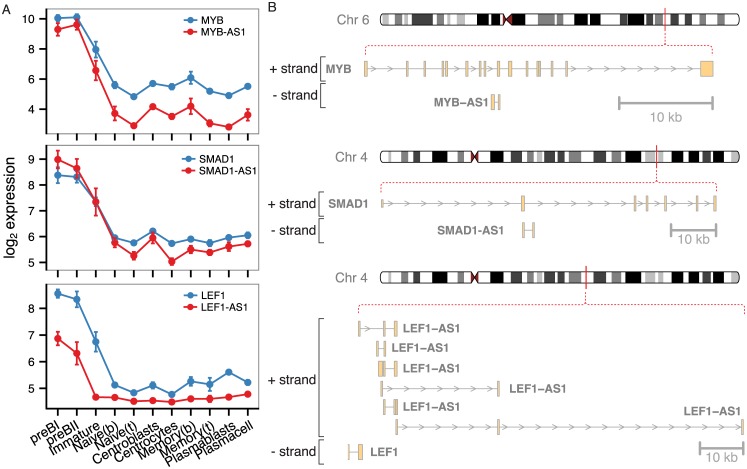
Antisense RNAs in the brown module center. A) Expression profiles and B) genomic organization of highly connected sense-antisense pairs from the brown module.

**Fig 7:**
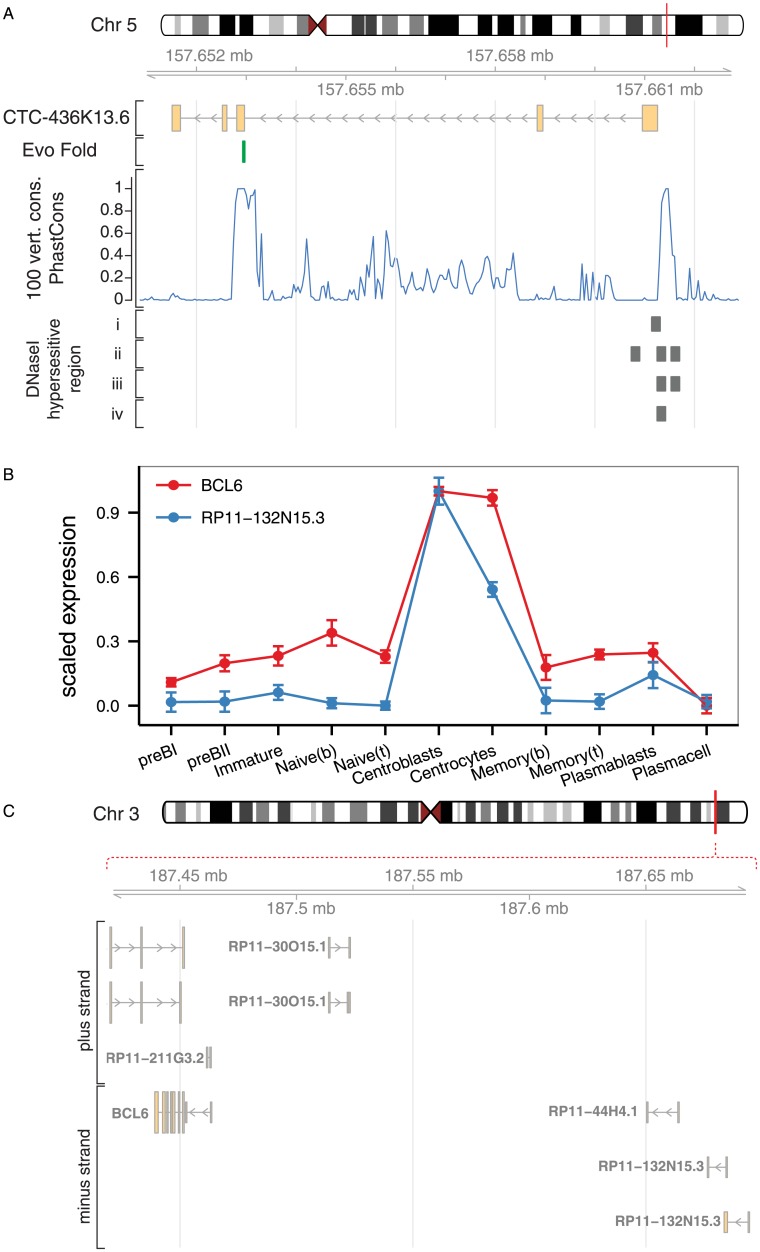
Highly connected lincRNAs in the brown and yellow modules. A) Genome browser plot for the highly connected lincRNA CTC-436k13.6 in the brown module. The EvoFold track shows position of a highly conserved RNA secondary structure that overlaps the exon-intron boundary. PhastCons scores show conservation calculated from a 100 species genome-wide multiple sequence alignment. DNaseI hypersensitive region tracks show data from i) CD20+ B-cells, ii) CD14+ monocytes, iii) CD34+ hematopoietic progenitor cells, and iv) Jurkat cell line. B) Expression profiles of RP11-132N15.3 and the nearby BCL6 gene. C) Visualization of the genomic region containing RP11-132N15.3 and BCL6.

The hub genes of the brown module are shown in Fig 5A. Consistent with the notion that highly connected genes are important in networks, we find surrogate light chain (VPREB1), RAG2, and DNTT, which are important for generating diversity at the junctions of rearranged Ig heavy genes, as well as transcription factors LEF1 and MYB to be hub genes. Interestingly, we identified several lncRNAs at the center of this module, including antisense transcripts to transcription factors with well-known roles in early B-cells (MYB—MYB-AS1, SMAD1—SMAD1-AS1, and LEF1—LEF1-AS1) and a lincRNA called CTC-436K13.6. While MYB-AS1 and SMAD-AS1 are simple transcripts each with two exons, the LEF1-AS1 has multiple exons and encodes several transcript variants and only one of the isoforms is a true antisense RNA (Fig 6). Antisense transcripts are an interesting subclass of lncRNAs that can exert regulatory effects directly on their sense transcript, in *cis* on neighboring genes, and even in *trans* on distal genes, co-transcriptionally or post-transcriptionally (reviewed in ref. [13]). The role of such antisense transcripts during B-cell development is currently unknown, but their central position in the brown module suggests important functions in early B-cell development. In addition, the central part of the brown module contains a highly connected lincRNA (CTC-436K13.6), which is located on chromosome 5, between genes CLINT1 and EBF1. Both of these genes are expressed at various stages of B-cell development, but none of them show an expression profile similar to CTC-436K13.6. The EBF1 gene encodes the transcription factor Early B-cell Factor 1, which is essential for establishing a transcription factor network ensuring B-cell line commitment [59]. Results from the ENCODE project identifies the 5’ end of CTC-436K13.6 and its upstream region as DNaseI hypersensitive in a variety of cell types, including CD34^+^ hematopoietic progenitor cells mobilized from a donor treated with G-CSF, CD20^+^ B cells, CD14^+^ monocytes, and Jurkat cells, but not in common cell lines, such as HepG2, HeLa-S3, and Huh7 (Fig 7A). Active regulatory regions and especially promoters tend to be DNaseI-sensitive, which provides further evidence that the lincRNA is transcribed in cells of hematopoietic origin. To examine the sequence conservation of this lincRNA, we used PhastCons scores calculated from multiple alignment of 100 vertebrate species available through the UCSC genome browser [60,61] and observed that exon 3 and the surrounding intronic sequences as well as the promoter region immediately upstream of the lincRNA are all well-conserved. Furthermore, we observed that the junction between the 2^nd^ intron and 3^rd^ exon is spanned by a conserved stem-loop structure [62], suggesting that this lincRNA could be subject to alternative splicing [63]. It has previously been reported that lincRNA homology is often restricted to short, highly conserved sequences [2] and that lncRNA promoters often show higher conservation than protein-coding gene promoters [64]. However, despite the fact that certain elements of this lincRNA overlap with highly conserved genomic regions and the fact that CTC-436K13.6 falls in a syntenic block, there are currently no reported orthologues.

Apart from the lncRNAs discussed above, we identified several additional lncRNAs that are part of the brown module (S1 Table). For each lncRNA we report correlation (both Pearson’s and Spearman’s) to the module eigengene, which can help identify whether the lncRNA has an expression profile that is similar to the eigengene (i.e. expressed in early B-cell) or whether it is absent from early B-cells and expressed later during B-cell development.

#### Proliferative stages of B-cell development

Genes in the turquoise module exhibit highest expression in pre-B1, pre-B2 cells, as well as centroblasts and to a lesser extent in centrocytes (Fig 4B), or the opposite expression profile (i.e. down-regulated or absent in pre-B1, pre-B2, centroblasts, and centrocytes, S2 Fig). The module members show highly significant overrepresentation of genes involved in mitotic cell cycle related processes (Table 1 and S2 Table) consistent with the fact that both pre-B cells and germinal center centroblasts are actively proliferating cells [65]. The genes at the center of the module (Fig 5B) are all tightly connected, and many of the hub genes are well-characterized key players in cell cycle processes. Several lncRNAs show strong and highly significant correlation to the turquoise module eigengene (S1 Table). The lincRNA CRNDE is part of the turquoise module, but since it also exhibits moderate expression in plasmablasts and plasma cells, it is not centrally located in this module. Of note, CRNDE has been found to be up-regulated in several tumors, particularly neoplasms of blood and brain [66,67]. In addition, analysis of published array data on differentiating CD4+ T-cells has indicated that CRNDE expression decreases as cells differentiate from a progenitor stage to naive T-cells, suggesting that CRNDE is generally expressed during lymphocyte development [67,68]. A study of lincRNAs interacting with chromatin-modifying complexes showed direct interactions between CRNDE and PRC2 as well as CoREST, and that there is an overlap in genes affected by siRNA-mediated knockdown of CRNDE and PRC2, implying that CRNDE is involved in chromatin modification [69]. Interestingly, a recent study has linked CRNDE to regulation of central metabolism by showing that it promotes metabolic changes that switch cancer cells to aerobic glycolysis [70]. Many cells use aerobic glycolysis during rapid proliferation [71] and the expression of CRNDE in primarily pre-B1, -B2, and centroblasts is consistent with its newly identified role as a metabolic regulator.

#### The germinal center

The yellow module consists of genes that are primarily expressed in centrocytes and centroblasts or are absent or down-regulated in the germinal center (Fig 4B and S2 Fig). GO analysis shows overrepresentation of genes assigned to ‘cellular response to stimulus’, ‘developmental process’, and ‘regulation of G-protein coupled receptor protein signaling pathway‘ (Table 1 and S2 Table). The latter is used to annotate 7 different genes, including RGS13, which is important for regulating the responsiveness of B-cells to CXCL12 and -13 in the germinal center [72]. The module hub genes (Fig 5C) include AICDA and SERPINA9, which have been found to be expressed exclusively in germinal center B-cells and malignant cells derived from germinal center B-cells [73]. Similar to the brown module, we identified several lincRNAs among the hub genes (LINC00487, LINC00877, and RP11-203B7.2) (Fig 5C). Interestingly, we also found a lincRNA, designated as RP11-132N15.3, outside the immediate module center, which is predominantly expressed in centroblasts and to some extent in centrocytes (Fig 7B). It is encoded on chromosome 3 approximately 240 kilobases upstream of BCL6 (Fig 7C). BCL6 is a master regulator of the germinal center reaction and modulates target genes in several different signaling pathways. These work together to increase the tolerance for DNA damage allowing genetic modifications of immunoglobulin genes, impair premature activation of B cells, and block terminal differentiation of B cells to enable the development of high affinity antibodies [74]. The genomic region between BCL6 and RP11-132N15.3 contains another lincRNA, which was not considered in this study due to low expression levels, however, analysis of the unfiltered data revealed an expression trend similar to RP11-132N15.3 (data not shown). To extend transcription profiling of this lincRNA, we explored phase 1 and phase 2 CAGE data from the FANTOM5 project and identified expression of RP11-132N15.3 in pools of normal human tonsil, corroborating our findings, and furthermore in the Burkitt's lymphoma cell lines RAJI and DAUDI, as well as hairy B cell lymphoma cell line MLMA. The four CAGE libraries showing expression of RP11-132N15.3 corresponded to 0.2% of all libraries analyzed, suggesting that this lincRNA is highly tissue-specific [75–77]. CAGE tags for the intervening lincRNA could also be identified in the same samples, albeit at lower levels, which is in agreement with our expression data.

## Conclusion

While the intrinsic high levels of genomic instability during stages of B-cell development are necessary for the development of high affinity B-cells, they also carry an inherent risk of errors that can drive malignant transformation. Translocation, amplification, deletion, and mutation events can all lead to aberrant expression of factors that control proliferation, differentiation, and apoptosis [78]. Indeed, several malignant lymphomas have been found to originate from distinct stages of normal B-cell development, in particular the germinal center B-cells, and studies have revealed that events and factors that are of key importance to normal B-cell development are also important in lymphomagenesis (reviewed in refs [79–82]). Recent data implies that lncRNAs are important regulators of highly diverse biological processes and that their dysregulation can be linked to the pathogenesis of cancer [83]. Thus, the identification of lncRNAs associated with distinct stages of B-cell development presented in this work will not only be an important resource for future work on exploring the molecular mechanisms underlying normal B-cell lymphopoiesis, but will also provide the basis for understanding the roles of lncRNAs in the pathogenesis and progression of B-cell malignancies.

## Supporting Information

S1 Fig Comparison of sample clustering based on specific gene biotype subtypes. Sample clustering based on expression of protein-coding genes compared with sample clustering based on expression of lincRNAs (A) or antisense RNAs(B).
(PDF)

S2 Fig Expression profiles of all identified gene co-expression modules. For each module, the module eigengene expression profile is shown below a heatmap of all genes in the module.
(PDF)

S1 Table Long noncoding RNAs associated with the identified gene co-expression modules. LncRNAs associated with the identified modules are listed along with Ensembl annotations (ID, biotype, and genomic coordinates), summary of coding potential analysis (number of transcripts: number of transcript variants transcribed from gene; cpat.mean: average coding probability for all transcripts; cpat.max: coding probability of transcript with highest coding potential; cpat.range: range in coding probabilities of all transcripts), and correlation of gene expression to respective module eigengene (spear + p.val_s: Spearman’s correlation and P value; pear + p.val_p: Pearson’s correlation and P value).
(XLSX)

S2 Table Overrepresented Gene Ontology terms in the identified gene co-expression modules. The top 10 overrepresented GO terms (p < 0.01) in each of the identified gene co-expression module are listed.
(XLSX)
