# Supplementary figures and images for: Long Noncoding RNA Expression during Human B-Cell Development
Source: PLoS One. 2015 Sep 22;10(9):e0138236. doi: 10.1371/journal.pone.0138236 (PMC4578992; doi:10.1371/journal.pone.0138236)

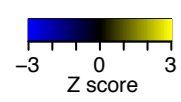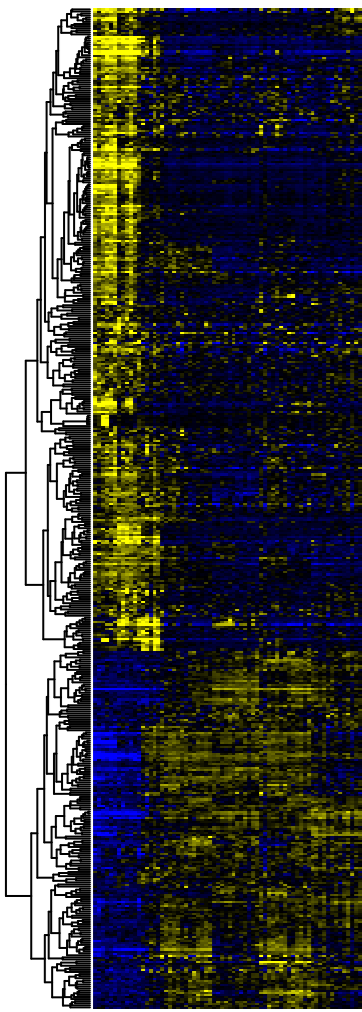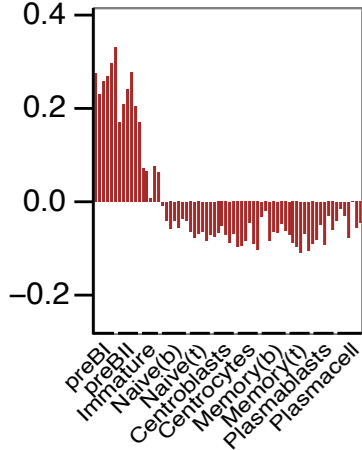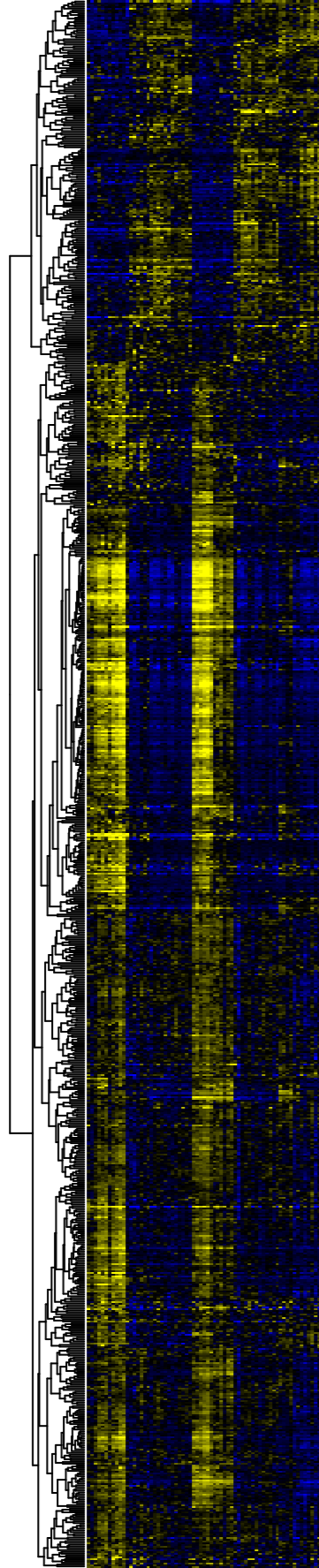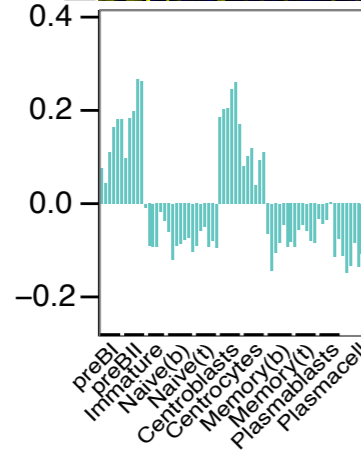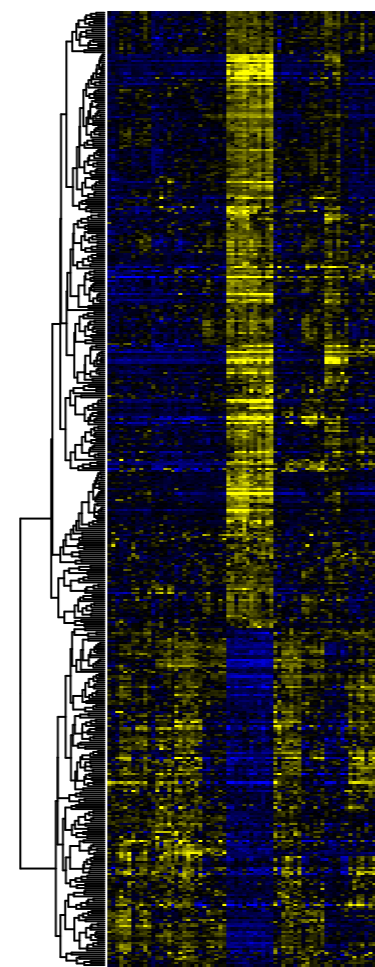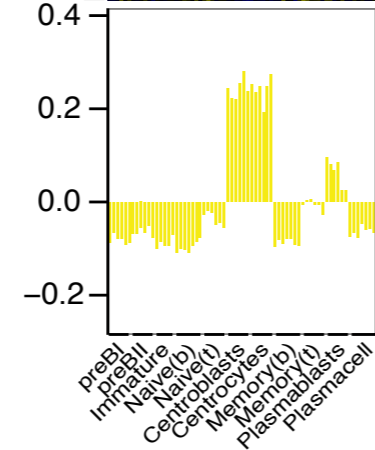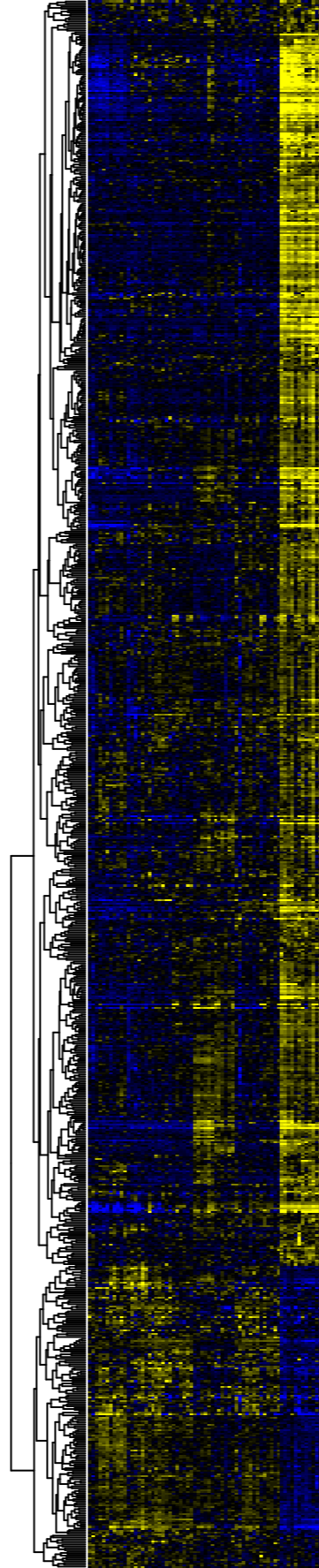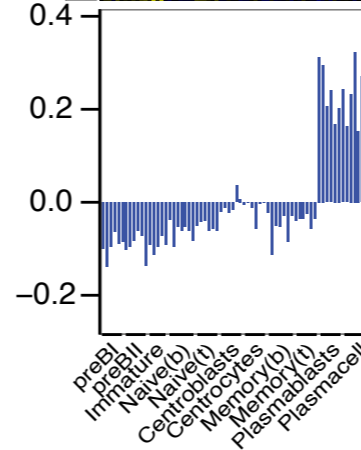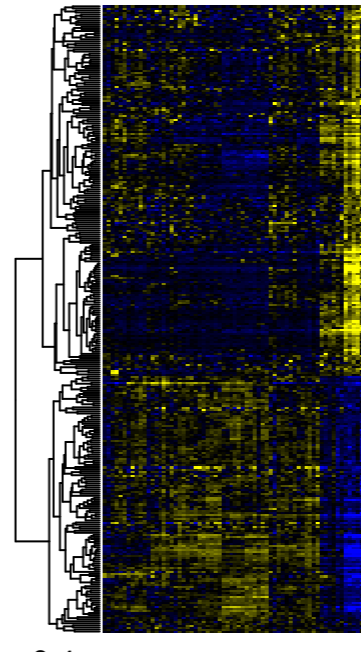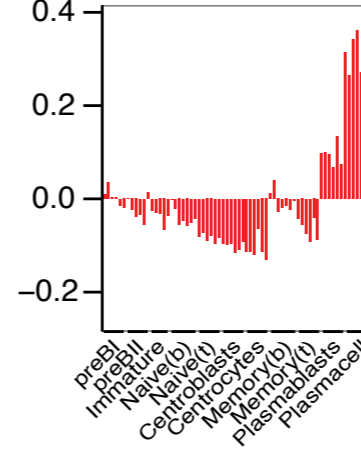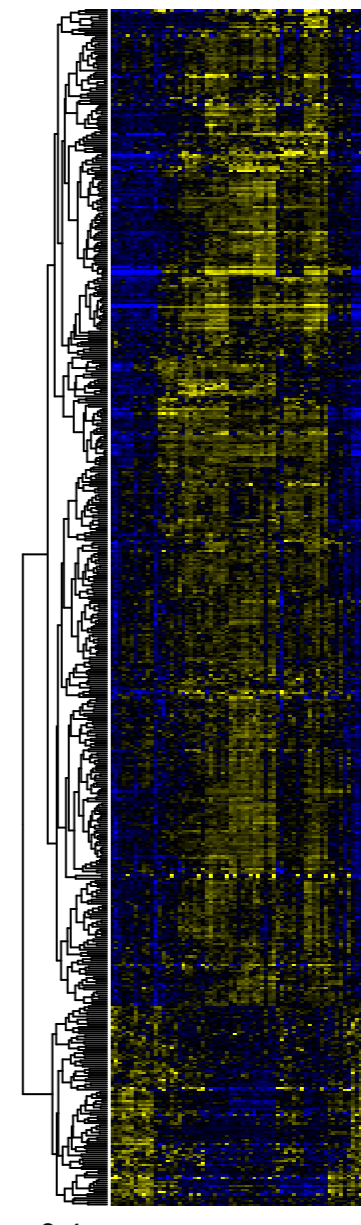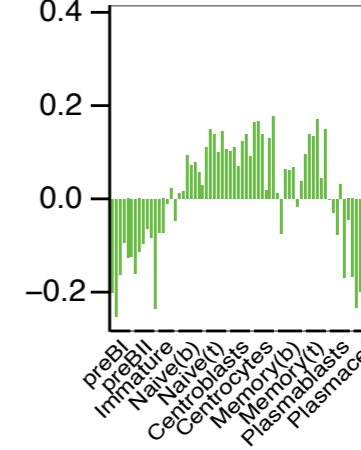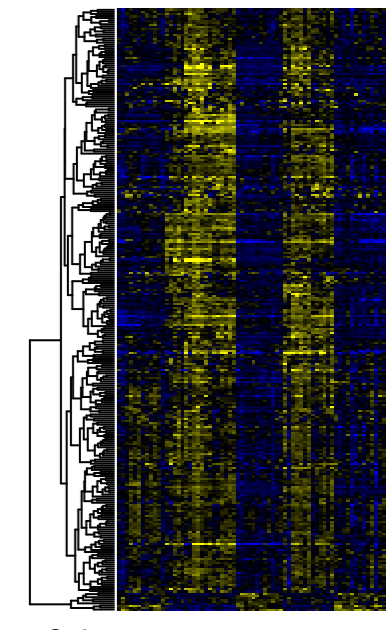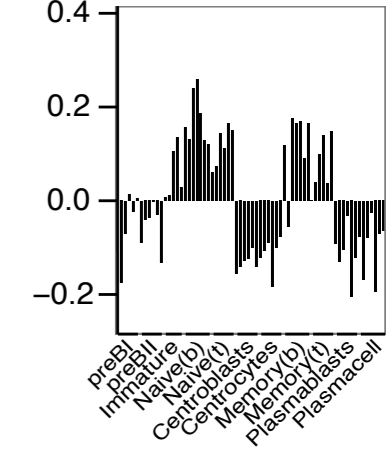

Supplement: S2 Fig — For each module, the module eigengene expression profile is shown below a heatmap of all genes in the module. (PDF) [file pone.0138236.s002.pdf]
