# Supplementary material for: Long Noncoding RNA Expression during Human B-Cell Development
Source: PLoS One. 2015 Sep 22;10(9):e0138236. doi: 10.1371/journal.pone.0138236 (PMC4578992; doi:10.1371/journal.pone.0138236)
Supplement: S1 Fig — Sample clustering based on expression of protein-coding genes compared with sample clustering based on expression of lincRNAs (A) or antisense RNAs(B). (PDF) [file pone.0138236.s001.pdf]

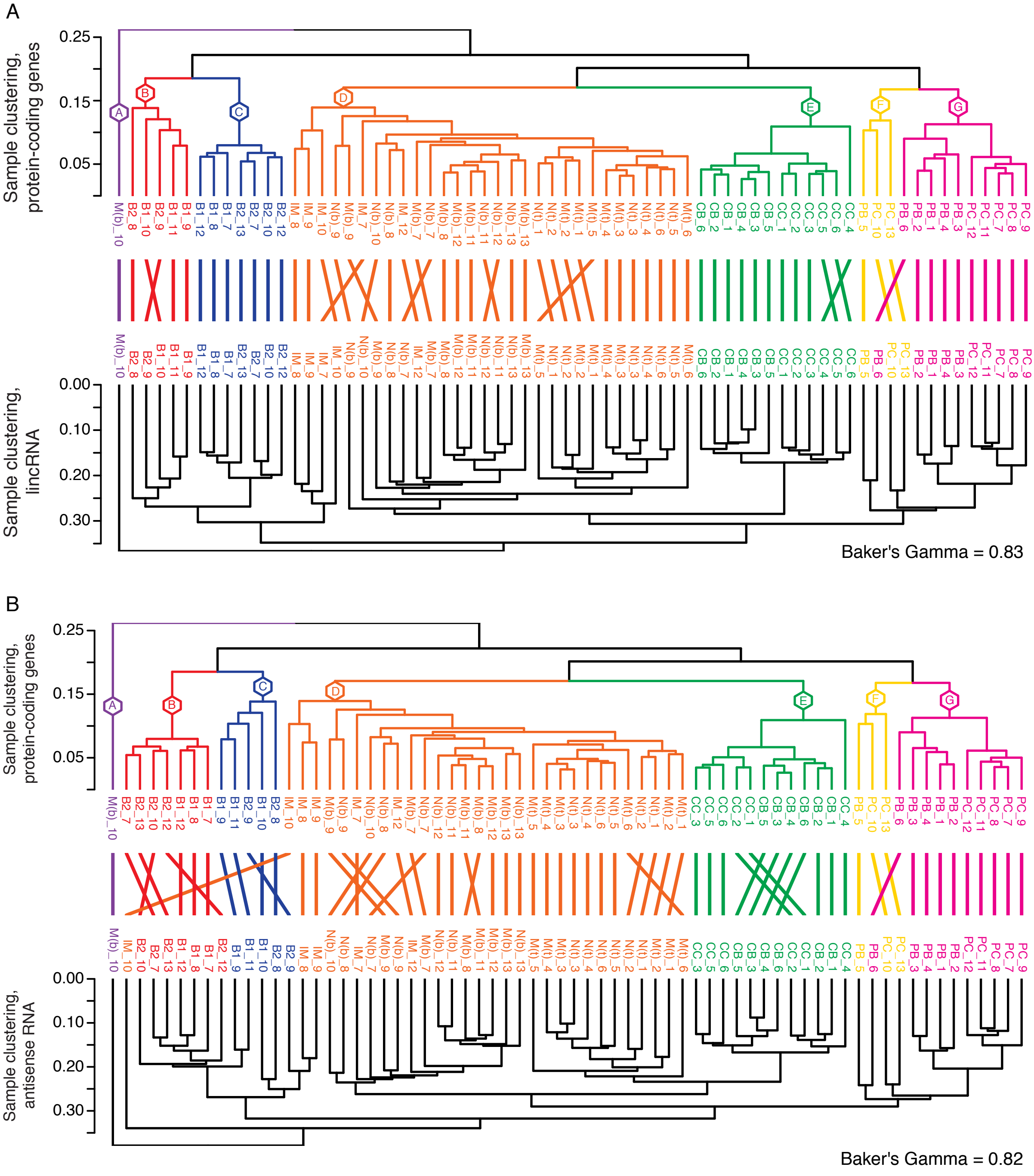

A
Sample clustering, protein-coding genes
0.25
0.15
0.05
A
B
C
D
E
F
G
Sample clustering, lincRNA
0.00
0.10
0.20
0.30
Baker's Gamma = 0.83
B
Sample clustering, protein-coding genes
0.25
0.15
0.05
Sample clustering, antisense RNA
0.00
0.10
0.20
0.30
Baker's Gamma = 0.82
